# Using Chinese Version of MYMOP in Chinese Medicine Evaluation: Validity, Responsiveness and Minimally Important Change

**DOI:** 10.1186/1477-7525-8-111

**Published:** 2010-09-30

**Authors:** Vincent CH Chung, Vivian CW Wong, Chun Hong Lau, Henny Hui, Tat Hing Lam, Lin Xiao Zhong, Samuel YS Wong, Sian M Griffiths

**Affiliations:** 1 School of Public Health and Primary Care, Chinese University of Hong Kong. Address: 2/F, School of Public Health, Prince of Wales Hospital, Shatin, Hong Kong SAR, China; 2 Chinese Medicine Department, Hospital Authority Head Office. Address: 3/F, Block C, Buddist Hospital, 10 Heng Lam Street, Lok Fu, Kln, Hong Kong SAR, China; 3 Yan Chai Hospital cum The Chinese University of Hong Kong Chinese Medicine Training and Research Centre. Address: 2/F, Block E, Yan Chai Hospital, 7-11, Yan Chai Street, Tsuen Wan, NT, Hong Kong SAR, China

## Abstract

**Background:**

Measure Yourself Medical Outcome Profile (MYMOP) is a patient generated outcome instrument applicable in the evaluation of both allopathic and complementary medicine treatment. This study aims to adapt MYMOP into Chinese, and to assess its validity, responsiveness and minimally important change values in a sample of patients using Chinese medicine (CM) services.

**Methods:**

A Chinese version of MYMOP (CMYMOP) is developed by forward-backward-forward translation strategy, expert panel assessment and pilot testing amongst patients. 272 patients aged 18 or above with subjective symptoms in the past 2 weeks were recruited at a CM clinic, and were invited to complete a set of questionnaire containing CMYMOP and SF-36. Follow ups were performed at 2^nd ^and 4^th ^week after consultation, using the same set of questionnaire plus a global rating of change question. Criterion validity of CMYMOP was assessed by its correlation with SF-36 at baseline, and responsiveness was evaluated by calculating the Cohen effect size (ES) of change at two follow ups. Minimally important difference (MID) values were estimated via anchor based method, while minimally detectable difference (MDC) figures were calculated by distribution based method.

**Results:**

Criterion validity of CMYMOP was demonstrated by negative correlation between CMYMOP Profile scores and all SF-36 domain and summary scores at baseline. For responsiveness between baseline and 4^th ^week follow up, ES of CMYMOP Symptom 1, Activity and Profile reached the moderate change threshold (ES>0.5), while Symptom 2 and Wellbeing reached the weak change threshold (ES>0.2). None of the SF-36 scores reached the moderate change threshold, implying CMYMOP's stronger responsiveness in CM setting. At 2^nd ^week follow up, MID values for Symptom 1, Symptom 2, Wellbeing and Profile items were 0.894, 0.580, 0.263 and 0.516 respectively. For Activity item, MDC figure of 0.808 was adopted to estimate MID.

**Conclusions:**

The findings support the validity and responsiveness of CMYMOP for capturing patient centred clinical changes within 2 weeks in a CM clinical setting. Further researches are warranted (1) to estimate Activity item MID, (2) to assess the test-retest reliability of CMYMOP, and (3) to perform further MID evaluation using multiple, item specific anchor questions.

## Background

Given the fundamental differences between allopathic medicine and traditional, complementary and alternative medicine (TCAM), conventional approaches in clinical research may not be directly applicable to the evaluation of TCAM [1-3]. One of the major challenges in designing TCAM clinical study is the need in adopting appropriate outcome measures that is compatible with the complexity of TCAM interventions [4,5]. Understanding the effect of TCAM from patients' own perspective is a plausible starting point for evaluation [6,7]. This mandates the development of patient centred measurement tools that are able to balance the requirement of capturing TCAM specific effects, as well as maintaining optimal psychometric properties. Measure Yourself Medical Outcome Profile (MYMOP) is an exemplar tool in this regard as it is a brief validated instrument that measure changes based on patients' subjective preference and assessment [8]. During MYMOP administration, patients are invited to nominate one or two symptoms which are especially of concern to them, together with one daily activity that is being limited by these symptoms. The respondent then rates these items, plus a question on general wellbeing, on a 7 point scale ranging from "as good as it could be" to "as bad as it could be". A profile score can be calculated by averaging individual item score.

As an evaluative tool, MYMOP has been found to be applicable in both allopathic and TCAM clinical settings [9], with a particular strength in being more responsive than SF-36 [8]. Qualitative evaluation of MYMOP suggested that there is a good concordance between TCAM patients' personal account of clinical changes and the quantified description by MYMOP [10], despite its limitations in overcoming response shifts and in capturing changes in new or episodic symptoms over time[11,12]. MYMOP has been increasingly adopted in the evaluation of TCAM programs in the past decade [13-17]. In China, a clinical efficacy driven approach for evaluating Chinese medicine (CM) has been advocated as a research priority, and this calls for conducting more rigorously designed CM trials with appropriate outcomes [3]. Nevertheless, few patient centred clinimetric tools for TCAM evaluation are currently available to Chinese researchers as most of them are developed in English [18]. In this study, we aim to assess the validity, responsiveness and minimally important change of a Chinese version of MYMOP, in a CM clinical setting in China.

## Methods

### Forward - Backward - Forward Translation of MYMOP

In translating MYMOP from English to Chinese, we followed guideline developed by Beaton and colleagues [19]. First, forward translation were performed by one investigator with clinical and health service research method training (VC), and one professional translator (T1) without healthcare background. Two forward translations of MYMOP were hence generated (MYMOP - Forward1 and MYMOP - Forward2). By discussion between VC, LCH and T1, a single consensus based Chinese translation was produced (MYMOP - Forward3). Second, MYMOP - Forward3 was back translated into English by two Chinese translator (T2 and T3) residing in the U.S. Two back translated English versions (MYMOP - Backward1 and MYMOP - Backward2) were generated. SG and SW, who are academic clinicians in public health and primary care, discussed discrepancies in the two backward translations and produced a single harmonised version of back translation (MYMOP - Backward3). Third, VC, LCH and another professional translator (T4) worked collaboratively and translated MYMOP - Backward3 into Chinese (MYMOP - Forward4).

### Pilot testing of translated version

The semantic and conceptual equivalence between original MYMOP and MYMOP - Forward4 was evaluated by an expert panel consisting of 15 healthcare professionals with diverse backgrounds. One to one cognitive debriefing interviews were conducted amongst panel members and their comments on each item were noted. VC, LCH and SW analysed these qualitative comments and performed amendments to the items. Feedback about the changes were then sought from all expert panel members, and a new consensus based version was generated (MYMOP - Forward5). Finally, MYMOP - Forward5 was piloted in 28 patients who had experience in using allopathic medicine as well as CM. Each patient was invited to complete the questionnaire, and was interviewed about the meaning of each item following a cognitive debriefing approach. Findings from the patient pilot were analysed by the authors and a final Chinese version was produced (CMYMOP). Besides MYMOP, our translation and pilot testing process also included the Chinese adaptation of a question on patient perceived global change, which was used in the original MYMOP validation (How would you rate your condition now compared to the last time you measure it?: Much better/A little better/About the same/A little worse/Much worse) [8]. In this study, this question is used as an anchor question for estimating minimal important difference of CMYMOP scorings.

### Setting and sampling

We performed a single group longitudinal study from July to December 2008 with consecutive patients who attended the Yan Chai Hospital cum The Chinese University of Hong Kong Chinese Medicine Training and Research Centre (YC CMCTR), operated by Yan Chai Hospital Board in tripartite collaboration with the Hospital Authority and the Chinese University of Hong Kong. YCCMCTR provides Chinese herbal medicine, acupuncture and therapeutic massage services. At enrolment, patients were informed on study purpose, and were assessed for study eligibility by a CM practitioner (CMP) before consultation. Inclusion criteria were: (1) aged 18 or above, (2) able to provide written Informed consent, (3) able to read and write Chinese without assistance, (4) self reported to suffer from at least one specific symptoms for in the last 14 days. Exclusion criteria were: (1) those reported no specific, subjective, symptomatic complaint in the past 14 days, and (2) patients who refuse to provide consent or telephone number for follow up.

### Data collection and follow up

After consultation, eligible patients were invited to complete a questionnaire package containing CMYMOP, previously validated Hong Kong Chinese version of SF-36[20], as well as health and demographic questions. Follow up assessments using CMYMOP, SF-36 and patient perceived change question were performed at 2^nd ^and 4^th ^week post consultation, either via face to face or telephone interview. In both formats, reminders on baseline CMYMOP Symptoms 1, Symptom 2 and Activities entries were given, but previous scorings were concealed. For time frame of reference, we used "past 7 days" at baseline, and "past two weeks" for follow-ups. The time frame of reference for follow ups was one week longer than the original English version. This change is grounded on our pilot results, which suggested that many patients found it difficult to isolate their subjective experience in the past 7 days when they performed follow up after two weeks. A trained CMP assisted patients in all episodes of data collection, but patients were strongly encouraged to follow their own perspective when scoring each CMYMOP and SF-36 items. A small gift was given to each enrolled patient as an incentive. Ethics approval was obtained from Chinese University of Hong Kong Clinical Research Ethics Committee.

### Data analysis

Criterion validity of CMYMOP was assessed by the strength of correlation between CMYMOP and SF-36 scores at baseline. Based on previous study which showed low to moderate correlation between MYMOP and SF-36 scorings, the Pearson product-moment correlation coefficients between the two scores were hypothesized to range between 0.20-0.60 [8]. These coefficients were also expected to have a minus sign, as improvement is denoted by an increase in SF-36 scores, or a decrease in CMYMOP scores.

The statistical significance of change scores from baseline to two follow ups, as well as between follow ups were assessed by paired t-test. Following Norman et al.'s recommendation [21], responsiveness of CMYMOP was evaluated by calculating the Cohen's effect size (ES) of mean change scores at various intervals (baseline to 2^nd ^and 4^th ^week follow ups, and between 2^nd ^and 4^th ^week follow up). ES was calculated by dividing mean change scores with standard deviation (SD) of baseline mean scores. ES values of 0.20, 0.50, and 0.80 or greater was adopted to represent weak, moderate, and strong responsiveness [21].

We estimated minimal important difference (MID) and minimal detectable change (MDC) values of CMYMOP using anchor and distribution based approach respectively [22]. For MID, as we asked patient perceived change questions on two occasions (1. Early anchor: differences between baseline and 2^nd ^week follow up, and 2. Late anchor: differences between 2^nd ^week and 4^th ^week follow up), we were able to estimate MID using two anchors with different timeframe. For both anchors, MID values were regarded as the mean change scores of patients who indicated that they were "a little better" [23]. The corresponding MDC values were calculated by halving the SD of mean change scores [24]. All statistical analyses were performed by SPSS 15 software.

## Results

### Response and sample characteristics

At baseline, 539 were enrolled. At 2 weeks, 343 patients were followed up successfully (227 face to face interviews, 116 telephone interviews, response rate from baseline = 63.6%). 272 patients were followed up at 4 week (156 face to face interviews, 116 telephone interviews, response rate from baseline = 50.5%). The demographic and health characteristics of patients who completed all follow ups are presented in table 1.

**Table 1:** Participant characteristics

		n	%
Gender	Male	44	16.2
	
	Female	228	83.8

			

Age	<20	12	4.4
	
	20-29	37	13.6
	
	30-39	60	22.1
	
	40-49	61	22.4
	
	50-59	58	21.3
	
	60-69	26	9.6
	
	70-79	16	5.9
	
	>79	2	0.7

			

Highest Education Attained	Never received formal education/attended kindergarten	2	0.7
	
	Completed primary school	42	15.4
	
	Completed junior high school	60	22.1
	
	Completed high school	94	34.6
	
	Completed post-secondary education	29	10.7
	
	Completed undergraduate education	31	11.4
	
	Completed postgraduate education	14	5.1

			

Marital Status	Never married	84	30.9
	
	Married	155	57.0
	
	Widowed	8	2.9
	
	Divorced/Separated	21	7.7
	
	Refused to answer	4	1.5

			

Employment status	Employed full time	104	38.2
	
	Employed part time	29	10.7
	
	Unemployed	136	50.0
	
	Refused to answer	3	1.1

			

Current attendance to full time education course	Yes	23	8.4
	
	No	239	87.9
	
	Refused to answer	10	3.7

			

Self reported chronic disease status as diagnosed by a western allopathic doctor	Hypertension	45	16.5
	
	Diabetes	19	7.0
	
	Any heart diseases	16	5.9
	
	Stroke	11	4.0
	
	Asthma, emphysema, chronic bronchitis, or other chronic respiratory diseases	31	11.4
	
	Arthritis or any other chronic joint diseases	72	26.5
	
	Depression, anxiety disorder or any other psychiatric diseases	41	15.1

			

Health services utilization in the past month	Attended Chinese medicine consultation	222	81.6
	
	Attended western medicine consultation	134	49.3

### Criterion validity and responsiveness of CMYMOP

For criterion validity, all SF-36 domain and summary scores exhibited low to moderate correlation with CMYMOP profile score at baseline. All Pearson product-moment correlation coefficient values were negative and statistically significant, ranging from -0.314 to -0.454 (all p < 0.01, table 2).

**Table 2:** Criterion validity of CMYMOP: correlations between CMYMOP profile scores and SF-36 scores when questionnaires were first given

SF-36 Profile Score	Pearson correlation coefficient *
1. Physical Functioning	-0.345

2. Role, physical	-0.359

3. Bodily pain	-0.325

4. General Health	-0.447

5. Vitality	-0.454

6. Social functioning	-0.391

7. Role, emotional	-0.314

8. Mental health	-0.378

9. Physical Composite Summary	-0.368

10. Mental Composite Summary	-0.374

*All p < 0.001

For responsiveness between baseline and 4^th ^week follow up, ES of CMYMOP Symptom 1, Activity and Profile reached the moderate change threshold (ES>0.5), while Symptom 2 and Wellbeing reached the weak change threshold (ES>0.2). For baseline to 2^nd ^week follow up, ES of Activity reached moderate change threshold, and the remaining ES attained weak change threshold except Wellbeing. None of the ES between 2^nd ^and 4^th ^week follow up achieved weak or moderate threshold. Finally, ES of all SF-36 domains at all time frames failed to reach the moderate change threshold (Table 3).

**Table 3:** Mean changes and effect sizes of CMYMOP and SF-36 scores and effect sizes at baseline, 2^nd ^and 4^th ^week

Scale	Mean score at baseline (SD)	Baseline vs. Follow up at 2^nd ^week		2^nd ^week vs. 4^th ^week		Baseline vs. Follow up at 4^th ^week	
		
CMYMOP		Mean change in score* (SD)	ES	Mean change in score* (SD)	ES	Mean change in score* (SD)	ES
Symptom 1	3.574 (1.523)	-0.760 (1.719)	0.499	-0.193 (1.293)	0.126	-0.967 (1.859)	0.635

Symptom 2	3.597 (1.437)	-0.623(1.788)	0.433	-0.075 (1.390)	0.052	-0.696 (1.819)	0.485

Activity	3.689 (1.551)	-0.839 (1.615)	0.541	-0.118(1.286)	0.076	-0.972 (1.753)	0.627

Wellbeing	3.104 (1.439)	-0.222 (1.403)	0.154	-0.188(1.037)	0.130	-0.424 (1.483)	0.295

Profile	3.376 (1.281)	-0.488 (1.259)	0.381	-0.159(0.956)	0.124	-0.647 (1.401)	0.505

SF-36							

Physical Functioning	47.50 (9.287)	1.711 (5.605)	0.184	0.698 (4.207)	0.075	2.419 (5.779)	0.261

Role, physical	42.29 (11.35)	1.570 (8.781)	0.138	0.802 (7.167)	0.071	2.372 (9.265)	0.209

Bodily pain	44.30 (11.03)	2.841(9.546)	0.258	0.895 (9.454)	0.081	3.735 (9.542)	0.339

General health	36.90 (9.285)	0.675(6.328)	0.073	1.047 (5.847)	0.113	1.722 (6.369)	0.185

Vitality	44.91 (10.21)	0.870(7.884)	0.085	1.060(7.356)	0.104	1.930 (9.047)	0.189

Social functioning	41.58 (11.54)	2.086(8.809)	0.181	0.478(8.413)	0.041	2.564 (9.259)	0.222

Role, emotional	39.83 (13.32)	2.087(10.571)	0.157	0.246(9.303)	0.018	2.338 (11.809)	0.176

Mental health	41.75 (10.63)	0.317(8.507)	0.030	1.189(7.796)	0.112	1.505 (9.336)	0.142

Physical Composite Summary	44.85 (9.148)	1.876(5.707)	0.205	0.837(5.126)	0.092	2.743 (5.815)	0.300

Mental Composite Summary	40.41 (11.78)	0.997(8.548)	0.085	0.683(7.903)	0.058	1.660 (9.510)	0.141

Key: SD: Standard Deviation, ES: Cohen's Effect Size.

*Paired t test, all p ≤ 0.001

Table 4 shows baseline to 2^nd ^week CMYMOP mean change scores by varying degrees of patient perceived change. Distribution of mean change scores demonstrated the expected increment down the perceived global change gradient. This pattern resembled findings in the validation study of original English MYMOP [8]. However, for Activity item, our mean change scores for "a little better" and "about the same" were similar (-0.724 vs. -0.750). Therefore, we were unable to estimate MID for this item. For Symptom 1, Symptom 2, Wellbeing and Profile, their MID were 0.894, 0.580, 0.263 and 0.516 respectively (all expressed in absolute values). MDC from baseline to 2^nd ^week were 0.860 (Symptom 1), 0.894 (Symptom 2), 0.808 (Activity), 0.702 (Wellbeing) and 0.630 (Profile) respectively.

**Table 4:** Changes in mean CMYMOP scores from baseline to 2^nd ^week by categories of patient perceived change in clinical condition

	Mean (SD) change in score									
**Change rated by patients**	**Much better**	**n**	**A little better**	**n**	**About the same**	**n**	**A little worse**	**n**	**Much worse**	**n**

Symptom 1	-1.833 (1.781)	36	-0.894 (1.672)	141	-0.300 (1.529)	80	0.833 (1.193)	12	N/A	0

Symptom 2	-1.296 (2.284)	27	-0.580 (1.596)	81	-0.381 (1.821)	42	-0.125 (1.356)	8	N/A	0

Activity	-1.636 (1.590)	22	-0.724 (1.492)	87	-0.750 (1.832)	56	-0.571 (0.976)	7	N/A	0

Wellbeing	-0.611 (1.609)	36	-0.263 (1.346)	137	-0.114 (1.377)	79	0.667 (1.303)	12	N/A	0

Profile	-1.305 (1.541)	32	-0.516 (1.110)	136	-0.243 (1.280)	79	0.385 (0.832)	11	N/A	0

Key: SD: Standard Deviation, N/A: none of the patient reported "much worse"

**Figure 1:**
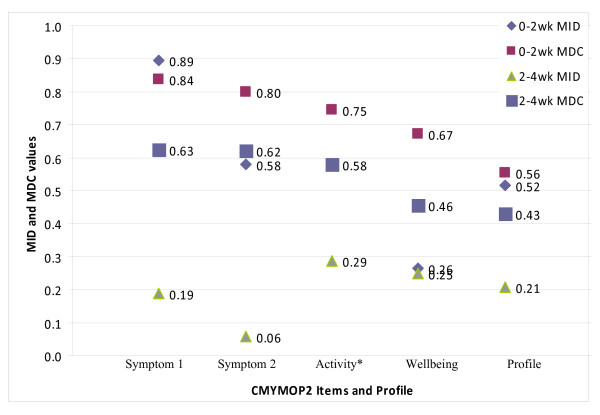
**Summary of Minimally Important Difference and Minimally Detectable**. Change Values of CMYMOP * MID of Activity item from 0-2 week anchor question was not estimated.

Result for 2^nd ^to 4^th ^week changes are presented in table 5. Distribution of all mean change scores demonstrated the expected increment down the perceived global change gradient. For Symptom 1, Symptom 2, Activity, Wellbeing and Profile scores, their respective MID values were 0.187, 0.056, 0.286, 0.250 and 0.206 respectively (all expressed in absolute values). MDC from 2^nd ^to 4^th ^week were 0.647 (Symptom 1), 0.700 (Symptom 2), 0.643 (Activity), 0.519 (Wellbeing) and 0.478 (Profile). All MID and MDC values are displayed graphically in Figure 1.

**Table 5:** Change in mean CMYMOP scores from 2^nd ^week to 4^th ^week by categories of patient perceived change in clinical condition

	Mean (SD) change in score									
**Change rated by patients**	**Much better**	**n**	**A little better**	**n**	**About the same**	**n**	**A little worse**	**n**	**Much worse**	**n**

Symptom 1	-0.892 (1.505)	37	-0.187 (1.250)	123	0.011(1.119)	88	0.333 (1.633)	15	N/A	0

Symptom 2	-0.696 (1.550)	23	-0.056 (1.241)	71	0.132 (1.359)	53	0.556 (1.944)	9	N/A	0

Activity	-0.769 (1.177)	26	-0.286 (1.157)	84	0.314 (1.241)	51	0.571 (1.742)	14	N/A	0

Wellbeing	-0.632 (1.364)	38	-0.250 (0.912)	116	0.047 (0.950)	85	0.267 (1.033)	15	N/A	0

Profile	-0.719 (1.163)	35	-0.206 (0.859)	119	0.063 (0.841)	85	0.500 (1.157)	14	N/A	0

Key: SD: Standard Deviation, N/A: none of the patient reported "much worse"

## Discussion

In this study, we conducted a Chinese adaptation of the English MYMOP questionnaire, and subsequently assessed the Chinese version's validity, responsiveness, MID and MDC values in a sample of Chinese patients using CM services.

### Validity and Responsiveness of CMYMOP

The criterion validity of CMYMOP was demonstrated by the negative correlation between CMYMOP Profile scores and all SF-36 domain and summary scores at baseline. Resembling validation result of the original English version [8], strength of correlation between the two scores was low to moderate. Only correlation coefficients between SF-36 General Health and Vitality domain scores, and CMYMOP Profile scores reached the conventional threshold of *r *≥ 0.45 [25]. Such observation maybe explained by the apparent construct difference between SF-36 and CMYMOP, in which the former aims to measure generic health related quality of life, and the later focuses on specific change of subjective symptoms. As an aspect of construct validity [26] and longitudinal validity [27], the responsiveness of CMYMOP and SF-36 also differed substantially in this study. At all comparison timeframes (baseline vs. 2^nd ^week, 2^nd ^vs. 4^th ^week, and baseline vs. 4^th ^week), ES of all SF-36 domain and summary scores did not demonstrate moderate change. On the contrary, ES of all CMYMOP scorings achieved moderate or small changes between baseline and 4^th ^week, implying a stronger responsiveness compared to SF-36.

While it is generally expected that longer follow up time is needed for capturing TCAM effect [28], our results showed that CMYMOP ES values at baseline to 2^nd ^week interval were much higher than that of the 2^nd ^to 4^th ^week interval. This suggests that most improvement was detected at first two weeks of CM treatment. Response shift at 4^th ^week follow up is a potential explanation for observing less improvement, as previous study has demonstrated that patients may raise their improvement expectation at later follow up time [12]. An alternative explanation is the strength of MYMOP in detecting improvement in acute conditions [8,29], in which this property subsequently portrayed a clustering of improvement at the first 2 weeks.

### MID and MDC of CMYMOP

Concentration of improvement at the first two weeks is also reflected in differences in MID values estimated from early (baseline to 2^nd ^week) and late (2^nd ^to 4^th ^week) anchors. Except for Wellbeing item in which MID from two anchors were similar, MID values for Symptom 1, Symptom 2 and Profile scores from early anchors were substantially higher than that from the late anchors. As mentioned in last paragraph, this may be a resultant effect of response shift, or CMYMOP's stronger ability in detecting acute change. In this case, the later explanation seems to be more plausible as our sample were attaching a lower expectation on CM treatment effect at 4^th ^week --- even a very small change in CMYMOP score (e.g. 0.1) was considered to be a slight improvement (table 5). From a reliability perspective, the usefulness of late anchor MID figures is doubtful as they are substantially lower than their corresponding MDC values. At the 2^nd ^to 4^th ^week timeframe, MDC figures ranged from 0.5 - 0.7, while MID ranged from 0.06 - 0.29 (Figure 1). Hence the question of whether a trivial mean change in CMYMOP score was attributed to patient perceived improvement, or to measurement errors, cannot be ascertained.

In fact, the problem of observing higher MDC compared to MID also appeared in our early anchor results, except for Symptom 1. Nevertheless, differences between the two sets of values are of lesser magnitude (Figure 1). These findings echo recent studies which showed how variations in sample characteristics and analysis methods contributed to large differences in minimally important change values [30]. Given the current emphasis in using anchor based method for establishing MID [22,23,30], a tentative conclusion based on early anchor MID values is preferred. However, as we were unable to estimate MID for Activity domain scores, the corresponding MDC value (0.702) may be used as a preliminary estimation.

Previous clinical studies using MYMOP as an outcome measure [15,31] have made no explicit discussion on MID, but gauged treatment effect size by referencing to conventional standard of mean change size typical for a seven points instrument (small change > 0.5; moderate change > 1.0, large change > 1.5) [32]. It is obvious that our tentative MID values are not compatible to this convention uniformly. While the MID for Profile score (0.516), Symptom 1 (0.894) and Symptom 2 (0.580) all resembled to the conventional small change threshold, MID for Wellbeing (0.263) was substantially lower. The question of why patients were attaching a lower expectation on Wellbeing as compared to Symptom 1 and 2 may partly be answered by our sample characteristics. As we exclusively enrolled patients with reported symptoms in the past 14 days, all included patients had an explicit intention in receiving treatments on specific symptoms. Thus, the relative importance of enhancing wellbeing could have been ranked lower when compared to that of alleviating the main symptoms. In view of such variations in patient expectations, further research is needed to examine the legitimacy of calculating CMYMOP Profile score by averaging item scores with equal weighting.

### Limitations of this study

This study has several weaknesses. First, we did not perform a test-retest reliability assessment due to difficulties in encouraging patients to repeat CMYMOP within a short period of time. This inhibited us from estimating MDC values using alternative methods like standard error of measurement (SEM) calculation, which is less dependent on data distribution[33,34]. Second, our patient perceived change question (anchor question) focused on global rating and thus ignored changes in specific CMYMOP items. In other words, our anchor question assumed all CMYMOP items to improve or deteriorate in the same directions, and the validity of this assumption requires further evaluation. Third, the response rates at 4th week follow up were mediocre and potential non-response bias cannot be ruled out. Forth, we adopted a dual approach of data collection by using both face to face and telephone interviews at follow ups. The effect of such variation on data quality requires further assessment, in which this would mandate an independent study with sufficient sample size that allows reliable comparison between the data collected by the two approaches. Finally, in response to our pilot results, we have changed the time frame of reference from the original "past 7 days" to "past 2 weeks" at follow, so as to facilitate our samples' understanding on the items. Similarly, a rigorous comparison is needed to assess the effect of such changes on the results.

## Conclusions

A Chinese version of MYMOP is developed using standard cultural adaptation methodology. In a CM clinical setting, CMYMOP is a valid and responsive instrument in capturing patient centred clinical changes within 2 weeks. Tentative MID values for Profile score ranged from 0.52 to 0.56. Further researches are warranted (1) to estimate Activity item MID, (2) to assess the test-retest reliability of CMYMOP, and (3) to perform further MID evaluation using multiple, item specific anchor questions.

## Competing interests

Data collection of study is funded by the Chinese Medicine Department, Hospital Authority Head Office and YCCMCTR.

## Authors' contributions

VC, VW and SG conceived the study and its design. LCH and SW designed and performed the statistical analysis. HH, LTH and LXZ monitored the translation and data collection process. VC drafted the manuscript with critical inputs from all authors. All authors read and approved the final manuscript.
